# Protein tyrosine phosphatase PTP-RR regulates corticosteroid sensitivity

**DOI:** 10.1186/s12931-016-0349-0

**Published:** 2016-03-24

**Authors:** Yoshiki Kobayashi, Kazuhiro Ito, Akira Kanda, Koich Tomoda, Anna Miller-Larsson, Peter J. Barnes, Nicolas Mercado

**Affiliations:** Airway Disease Section, National Heart and Lung Institute, Imperial College London, Guy Scadding Building, Royal Brompton Campus, Dovehouse Street, London, SW3 6LY UK; Airway Medicine, Department of Otolaryngology, Kansai Medical University, Osaka, Japan; AstraZeneca R&D Mölndal, Mölndal, Sweden

**Keywords:** Corticosteroid insensitivity, Glucocorticoid receptor, PP2A, PTP-RR, Severe asthma

## Abstract

**Background:**

We have recently reported that protein phosphate 2A (PP2A) inactivation resulted in increased phosphorylation of the mitogen-activated protein kinase (MAPK) c-Jun N-terminal kinase 1 (JNK1) and glucocorticoid receptors (GR) at Ser^226^, thereby reducing GR nuclear translocation and causing corticosteroid insensitivity in severe asthmatics. Protein tyrosine phosphatases (PTPs) are also known to be critically involved in the regulation of MAPKs, such as JNK and therefore potentially associated with GR function. The aim of study was to elucidate the involvement of MAPK-PTPs (PTP-RR, PTP-N5 and PTP-N7), which can dephosphorylate MAPKs, in the regulation of corticosteroid sensitivity.

**Methods:**

Corticosteroid sensitivity, GR nuclear translocation, phosphorylation levels of GR-Ser^226^, JNK1 and PP2A catalytic subunit (PP2A_C_)-Tyr^307^ and protein expression levels and activities of PTP-RR and PP2A_C_ were evaluated in U937 cells and/or peripheral blood mononuclear cells (PBMCs). Knock-down effects of MAPK-PTPs using siRNA were also evaluated.

**Results:**

Knock-down of PTP-RR, but not of PTP-N5 or PTP-N7 impaired corticosteroid sensitivity, induced GR-Ser^226^ phosphorylation and reduced GR nuclear translocation. Under IL-2/IL-4-induced corticosteroid insensitivity, PTP-RR expression, activity and associations with JNK1 and GR were reduced but PTP-RR activity was restored by formoterol. Also in PBMCs from severe asthmatic patients, PTP-RR and JNK1 expression were reduced and GR-Ser^226^ phosphorylation increased. Furthermore, PTP-RR was associated with PP2A. PTP-RR reduction enhanced PP2A_C_-Tyr^307^ phosphorylation leading to impairment of PP2A expression and activity.

**Conclusions:**

We demonstrated that with corticosteroid insensitivity PTP-RR fails to reduce phosphorylation of JNK1 and GR-Ser^226^, resulting in down-regulation of GR nuclear translocation. Reduced PTP-RR may represent a novel cause of corticosteroid insensitivity in severe asthmatics.

**Electronic supplementary material:**

The online version of this article (doi:10.1186/s12931-016-0349-0) contains supplementary material, which is available to authorized users.

## Background

Currently most patients with asthma are well controlled with the regular use of inhaled corticosteroids (ICS) with or without long-acting β_2_-adrenoceptor agonists (LABAs). Even though corticosteroids exert multifaceted anti-inflammatory effects, they are not disease-modifying and poorly effective in severe asthmatics who present a more corticosteroid insensitive phenotype [1]. Hence, elucidating the molecular mechanisms of corticosteroid insensitivity could lead a more effective therapeutic approach for severe asthma.

Defective function of glucocorticoid receptors (GR), such as decreased nuclear translocation, protein expression, ligand binding and binding to glucocorticoid-response elements (GRE) may be involved in corticosteroid insensitivity in severe asthma [2]. One possible cause of impaired GR nuclear translocation is the phosphorylation of GR by mitogen-activated protein kinases (MAPKs) [2–5]. In patients with severe asthma increased phosphorylation of GR and subsequent reduced nuclear translocation have been reported [2, 5, 6]. However, the molecular mechanisms of GR phosphorylation that lead to GR nuclear translocation have not yet been elucidated. Phosphorylation of Ser^203^ and Ser^211^ have been shown to induce GR activation [7–9] whereas Ser^226^ phosphorylation leads to inhibition of GR function [10–12]. Recently, we found that GR is hyperphosphorylated at Ser^226^ possibly via the action of c-Jun N-terminal kinase 1 (JNK1) in peripheral blood mononuclear cells (PBMCs) from severe asthmatics and in a corticosteroid insensitive PBMC model [13]. This is supported by previous findings that phosphorylation of Ser^226^ by JNK contributes to impairment of GR transcriptional activation [11] and GR shuttling [10]. Thus, GR-Ser^226^ phosphorylation might be involved in reduction of GR nuclear translocation.

Protein tyrosine phosphatases (PTPs) which regulate the level of tyrosine phosphorylation play a critical role in regulating signaling molecules such as mitogen-activated protein kinases (MAPKs) [14]. At least 107 PTPs are now recognized and they are classified into four groups based on the amino acid sequences of their catalytic domains [15, 16]. Tyrosine-specific classical PTPs which are classified into class I cysteine-based PTPs are further divided into two subgroups: receptor-like transmembrane PTPs; R1-R8 and non-transmembrane PTPs; NT1-NT9 [14]. The R7, one of receptor-like PTPs subgroup, termed ‘MAPK-PTPs’, consists of PTP-N5 [also known as striatal-enriched phosphatase (STEP)], PTP-N7 [also known as hematopoietic PTP (HePTP)] and PTP-RR [also known as PCPTP1 or STEP-like PTP (PTP-SL)]. PTP-N5, PTP-N7 and PTP-RR have all been reported to down-regulate MAPKs [17]. Among dual-specific phosphatases (DUSPs), another class I cysteine-based PTPs, MAPK phosphatases such as DUSP1 and DUSP4 are also involved in the regulation of MAPKs [16].

We have recently found that protein phosphatase 2A (PP2A), a serine/threonine phosphatase, regulates corticosteroid sensitivity by dephosphorylation of JNK1 and GR-Ser^226^, and PP2A located in the cell membrane/cytoplasm may be inactivated by phosphorylation of Tyr^307^ [13, 18], suggesting that other PTPs may be also involved in the regulation of corticosteroid sensitivity. We therefore hypothesized that reduction of MAPK-PTPs might also enhance JNK1 and GR-Ser^226^ phosphorylation resulting in impairment of GR nuclear translocation and corticosteroid sensitivity. In this study, we focus on ‘R7 PTPs (PTP-N5, −N7 and -RR)’, but not other MAPK phoshatases (DUSPs) which are mainly localized in the nucleus [19], and show that PTP-RR is down-regulated in PBMCs from severe asthmatics and in an *in vitro* corticosteroid insensitive cellular model. In addition, knockdown of PTP-RR (using siRNA) reduced GR nuclear translocation and corticosteroid sensitivity concomitantly with enhanced phosphorylation of JNK1 and GR-Ser^226^.

## Methods

### Reagents

3-(4,5-dimethylthiazol-2years)-2–5-diphenyltetrazolium bromide (MTT), dimethyl sulfoxide (DMSO), ICI-118551, salmeterol and salbutamol were purchased from Sigma-Aldrich (Poole, UK). Forskolin, a cAMP inducer (EC_50_ = 4 μM) (Calbiochem, Darmstadt, Germany) and MG-132 (Wako Pure Chemical Industries, Ltd., Osaka, Japan) were also purchased. Formoterol was obtained from AstraZeneca R&D Mölndal (Mölndal, Sweden). Recombinant human IL-2 and IL-4 were purchased from R&D Systems Europe (Abingdon, UK). The rabbit monoclonal antibody (Ab) to PP2A catalytic subunit (PP2A_C_), the rabbit polyclonal anti-GR (phospho S^226^) Ab, and the mouse monoclonal Ab to β-actin and TATA binding protein TBP were obtained from Abcam (Cambridge, UK). The rabbit polyclonal anti-GR Ab, the mouse monoclonal Ab to PP2A_C_ (phospho Try^307^), protein A/G plus-agarose and α-tubulin were obtained from Santa Cruz Biotechnology (Heidelberg, Germany). Rabbit polyclonal anti-phospho-SAPK/JNK and anti-SAPK/JNK Abs were obtained from Cell Signaling Technology (Danvers, MA). The rabbit polyclonal anti-PTP-RR Ab was purchased from Sigma Aldrich (Poole, UK) and LifeSpan BioSciences (Seattle, WA).

### Subjects

PBMCs obtained from 10 patients with severe asthma (SA) [20, 21] and 8 age-matched healthy volunteers (HV) from the Royal Brompton Hospital (London, UK) were isolated from whole blood using AccuSPIN (Sigma–Aldrich). As our previous data showed corticosteroid sensitivity in PBMCs from non-severe asthmatics was normal range [4, 5] without reduction of PP2A activity (unpublished data), we focused on the comparison between HV and SA in this study. Six of 10 subjects in SA and 5 of 8 subjects in HV were analyzed from samples used previously [13]. The characteristics of subjects are shown in Table 1. The severity of asthma was defined by GINA guideline [22]. All patients and healthy volunteers recruited for this study were never smokers. This study was approved by the local ethics committee of Royal Brompton and Harefield NHS Trust (07_Q0404_31) and written informed consent was obtained from each patient or volunteer.

**Table 1:** Characteristics Of Subjects

	Healthy volunteers (*n =* 8)	Severe asthmatics (*n =* 10)
Gender (M:F)	2:6	4:6
Age	49.9 ± 3.2	53.2 ± 4.9
FEV_1.0_ %pred.	90.7 ± 3.5	71.7 ± 5.6
FEV_1.0_/FVC	78.1 ± 1.5	69.1 ± 2.5
ICS	none	10/10
(mean dose)		^a^640 ± 72 μg
Oral prednisolone	none	6/10
(mean dose)		10.5 ± 3.0 mg
LABA	none	10/10

*ICS* inhaled corticosteroid, ^a^ fluticasone propionate equivalent dose, LABA: long-acting β_2_-agonist

### Cells

The human monocytic cell line U937 was obtained from the American Type Culture Collection (ATCC, Rockville, MD). Cells were cultured in complete growth medium (RPMI 1640; Sigma) supplemented with 10 % fetal bovine serum (FBS) and 1 % L-glutamine at 37 °C in a humidified atmosphere with 5 % CO_2_. Cell viability was assessed microscopically by trypan blue staining.

### Cell lysis, immunoprecipiation, and western blotting

Cell protein extracts were prepared using modified RIPA buffer (50 mM Tris HCL pH 7.4, 0.5–1.0 % NP-40, 0.25 % Na-deoxycholate, 150 mM NaCl with freshly added complete protease (Roche, Mannheim, Germany)), as described previously [13]. Phosphatase inhibitor (Active Motif, Rixensart, Belgium) and MG-132, a proteasome inhibitor, were used as needed. Nuclear extraction was performed using the Nuclear Extraction Active Motif kit (Active Motif). Protein concentration was determined using the Bio-Rad Protein Assay (Bio-Rad). Immunoprecipitation was conducted with anti-PTP-RR Ab (Abcam) or anti-PP2A Ab (Bethyl, Montgomery, TX). Protein extracts or immunoprecipitates were separated by SDS-PAGE (Invitrogen, Paisley, UK) and detected by Western Blot analysis using chemiluminescence (ECL Plus; GE Healthcare, Chalfont St. Giles, UK). β-actin expression was used as a control for protein loading as needed.

### Corticosteroid sensitivity

Cells were treated with dexamethasone (10^−6^ M or 10^−11^ M) for 45 min, followed by TNFα (10 ng/ml) stimulation overnight. The ability of dexamethasone to inhibit TNFα-induced CXCL8 release was determined in cell medium by sandwich ELISA according to the manufacturer’s instructions (R&D Systems Europe). IC_50_ values for dexamethasone on CXCL8 release were calculated using the computer program Prism 4.0 (GraphPad Software Inc., San Diego, CA) and were used as a marker for steroid sensitivity (Dex-IC_50_).

### Glucocorticoid receptor nuclear translocation

Cells were treated with dexamethasone (10^−7^ M) for 1 h. Nuclear and cytoplasmic GR were measured by Western Blot. TATA-binding protein (TBP) (for nuclear protein) or α-tubulin (for cytoplasmic protein) expression was used as a control for protein loading. The ratio of nuclear GR to TBP was used as an index of GR nuclear translocation.

### Protein phosphatase activity

Phosphatase activity was assayed by using the SensoLyte™ MFP Protein Phosphatase Assay Kit (AnaSpec, San Jose, CA) [13]. Immunopurified PTP-RR or PP2A were added into assay buffer (20 mM Tris–HCl pH 7.4, 100 mM NaCl, 1 mM EDTA, 1 mM DTT for PTP-RR; 40 mM Tris HCl pH 8.4, 34 mM MgCl_2_, 4 mM EDTA, 4 mM DTT for PP2A) and incubated with 3-O-methylfluorescein phosphate (MFP). Phosphatase activity was measured as a phosphatase-induced dephosphorylation which was monitored by measuring the fluorescence of MFP product. PTP-RR or PP2A immunoprecipitates were equally divided into two parts, with half used for phosphatase assay and the other half for target protein detection by Western Blot analysis. Phosphatase activity was normalized to the protein expression level calculated as band-density.

### RNA interference

PTP-N5, PTP-N7, PTP-RR and PP2A_Cα_ siRNAs and non-silencing scrambled control siRNA were purchased from QIAGEN (Crawley, UK). The siRNA sequences (0.5 μM) were transfected using an HVJ Envelope (HVJ-E) Vector Kit GenomONE-Neo (Ishikawa Sangyo Kaisha Ltd., Osaka, Japan) as described previously [13].

### Statistical analysis

Comparisons of two groups of data were performed using Mann–Whitney *U* test or paired *t*-test. Pearson’s or Spearman’s correlation coefficients were also calculated. Other data were analyzed by ANOVA with post *hoc* test adjusted for multiple comparisons (Bonferroni test or Newman-Keuls test), as appropriate. The difference was considered statistically significant if *P* < 0.05. Descriptive statistics were expressed as the mean ± SEM.

## Results

### PTP-RR reduction causes corticosteroid insensitivity

**Fig. 1:**
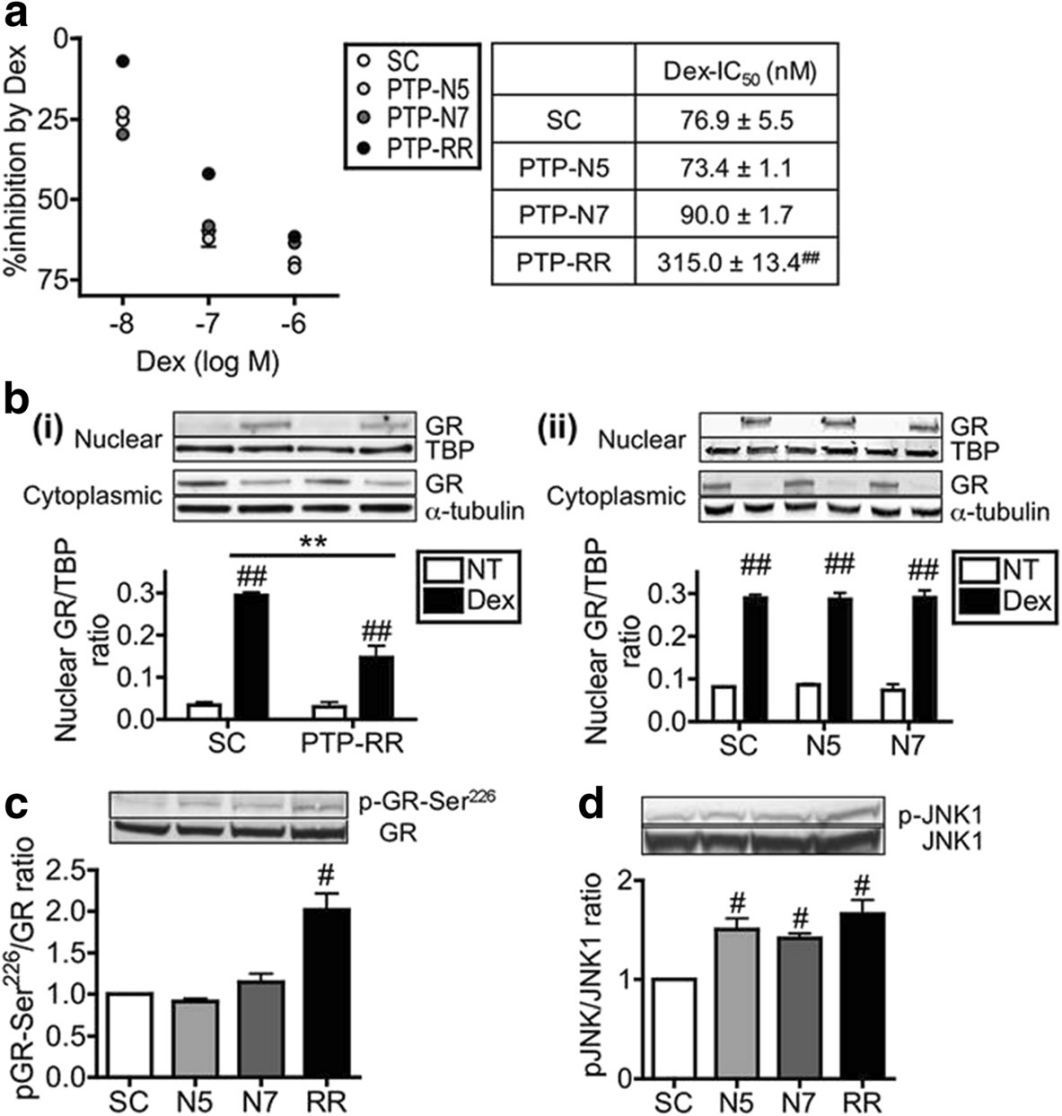
Effects of PTP-RR knockdown by siRNA. U937 cells were transfected with scramble control (SC), PTP-N5 (N5), PTP-N7 (N7) and PTP-RR (RR) siRNAs. **a** The ability of dexamethasone (Dex) to inhibit TNFα-induced IL-8 release was expressed as % inhibition by Dex (left panel) or Dex-IC_50_ (*right panel*). **b** GR nuclear translocation indicated as the ratio of nuclear GR to TBP (SC vs. RR siRNA in (i) and SC vs. N5 and N7 in (ii)). **c**, **d**, Phosphorylation levels of GR-Ser^226^ (**c**) and JNK1 (**d**). Data in **c** and **d** are expressed as fold change against SC. Values represent means of three (**a** and **d**) or four (**b** and **c**) experiments ± SEM: ^#^ *P* < .05, ^##^ *P* < .01 (vs. SC or NT in each group), ** *P* < .01 (as shown between two groups)

We first examined whether MAPK-PTPs (PTP-N5, PTP-N7 and PTP-RR) were involved in corticosteroid insensitivity in U937 cells. Western blotting analysis confirmed 30 % knockdown (KD) of PTP-RR, PTP-N5 and PTP-N7 in U937 cells and cell viabilities were more than 70 % (data not shown). Knockdown (KD) by siRNA of PTP-RR, but not of PTP-N5 or PTP-N7, resulted in reduction of dexamethasone inhibition of TNFα-induced CXCL8 release (left panel in Fig. 1a). This was further confirmed by a significant reduction in dexamethasone IC_50_ of 315.0 ± 13.4 nM in PTP-RR KD vs. 76.9 ± 5.5 nM in scrambled RNA control (right panel in Fig. 1a). Supporting these findings, PTP-RR KD, but not PTP-N5 KD or PTP-N7 KD, reduced dexamethasone-mediated GR nuclear translocation (Fig. 1b*(i) and (ii)*), together with an increase in phosphorylation of GR-Ser^226^, a biomarker of GR inactivation (Fig. 1c). Although all three MAPK-PTPs KDs increased JNK1 phosphorylation (Fig. 1d), only PTP-RR KD was associated with GR-Ser^226^ hyperphosphorylation (Fig. 1c).

### PTP-RR is down-regulated and dissociates from GR/JNK1 complex in corticosteroid insensitive cellular model

**Fig. 2:**
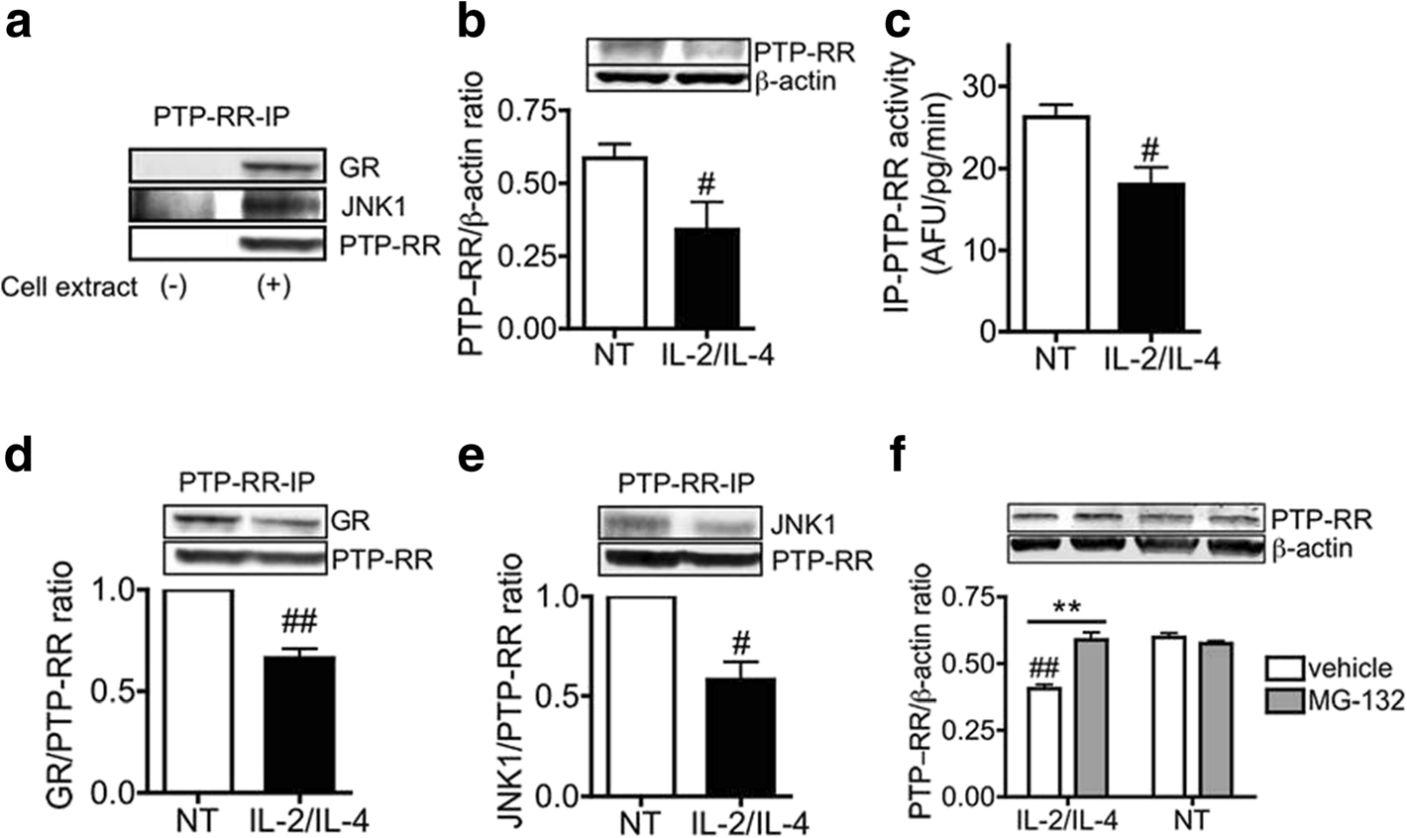
PTP-RR function under corticosteroid insensitive condition. **a** GR and JNK1 expression in PTP-RR-immunoprecipitates. **b**, **c**, **d**, **e**, **f**, U937 cells were exposed to IL-2/IL-4 for 48 h. PTP-RR protein expression (**b**) and activity (**c**) in whole cell extracts. GR (**d**) and JNK1 (**e**) protein expressions in PTP-RR-immunoprecipitates. Effect of MG-132 on PTP-RR expression (**f**). Data in (**d**) and (**e**) are expressed as fold change against non-treatment control (NT). Values represent means of four experiments ± SEM. ^#^ *P* < .05, ^##^ *P* < .01 (vs. NT) and ** *P* < .01 (as shown between two groups)

Immunoprecipitation of PTP-RR revealed that PTP-RR is associated with GR and JNK1, suggesting a possible role of this phosphatase in the function of corticosteroid dynamics [13, 18] (Fig. 2a). To further confirm the involvement of PTP-RR in corticosteroid insensitivity, we evaluated PTP-RR function with induced corticosteroid insensitivity. As showed previously [2, 23], co-stimulation of U937 cells with IL-2 (20 ng/ml) and IL-4 (10 ng/ml) (IL-2/IL-4) for 48 h induces corticosteroid insensitivity [4, 13]. Interestingly, IL-2/IL-4 reduced both PTP-RR protein expression and activity (Fig. 2b and c). In addition, IL-2/IL-4 dissociated PTP-RR from GR and JNK1 as shown by co-immunoprecipitation (Fig. 2d and e). Co-treatment with MG-132 (0.1 μM), a proteasome inhibitor, prevented IL-2/IL-4-induced PTP-RR reduction (Fig. 2f), suggesting that PTP-RR reduction via IL-2/IL-4-mediated degradation contributes, at least in part, to corticosteroid insensitivity.

### PTP-RR regulates PP2A function

**Fig. 3:**
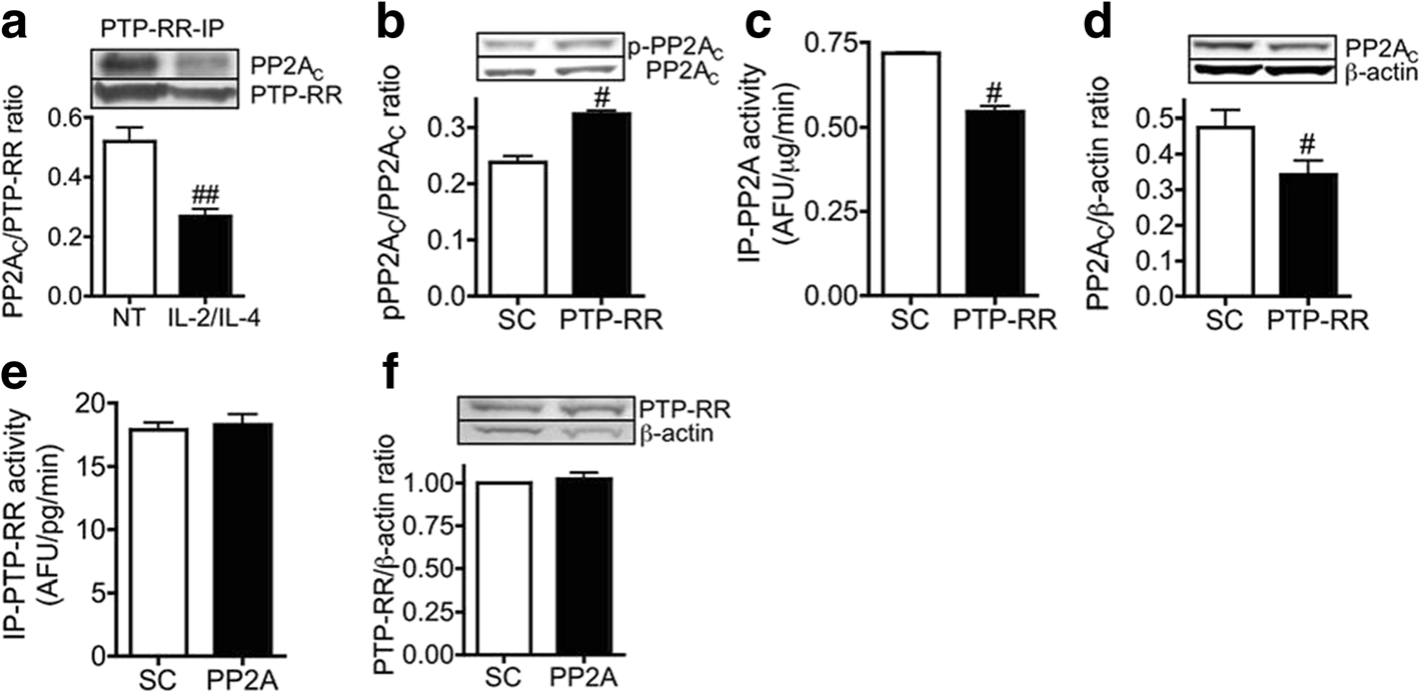
Regulation of PP2A by PTP-RR. **a** U937 cells exposed to IL-2/IL-4 for 48 h (or were not treated; NT). PP2A_C_ was detected in PTP-RR-immunoprecipitates. **b**, **c**, **d**, PP2A_C_-Tyr^307^ phosphorylation (**b**), PP2A activity (**c**) and PP2A_C_ protein expression (**d**) were analyzed in U937 cells transfected with scramble control (SC) or PTP-RR siRNAs. **e**, **f**, In U937 cells transfected with scramble control (SC) or PP2A siRNAs, immunopurified PTP-RR activity (**e**) and PTP-RR protein expression (**f**) were analyzed. Data in (**f**) is expressed as fold change against SC. Values represent means of four (**a**, **d** and **f**) or three (**b**, **c** and **e**) experiments ± SEM. ^#^ *P* < .05, ^##^ *P* < .01 (vs. non-treatment control; NT or SC)

Co-immunoprecipitation studies showed that PTP-RR is also associated with PP2A, suggesting a possible role of PTP-RR in the regulation of corticosteroid sensitivity by dephosphorylation of JNK1 and GR-Ser^226^ as shown previously for PP2A [13]. In fact, the association between PTP-RR and PP2A was reduced by 48 % after co-stimulation with IL-2/IL-4 (Fig. 3a). Additionally, PTP-RR KD enhanced PP2A catalytic subunit (PP2A_C_)-Tyr^307^ phosphorylation (Fig. 3b), which is required for down-regulation of PP2A activity [24]. In addition, reduced PP2A activity and protein expression were observed after PTP-RR KD (Fig. 3c and d). On the other hand, PP2A KD did not change PTP-RR activity or expression (Fig. 3e and f).

### PTP-RR is reduced in PBMCs from severe asthmatics

**Fig. 4:**
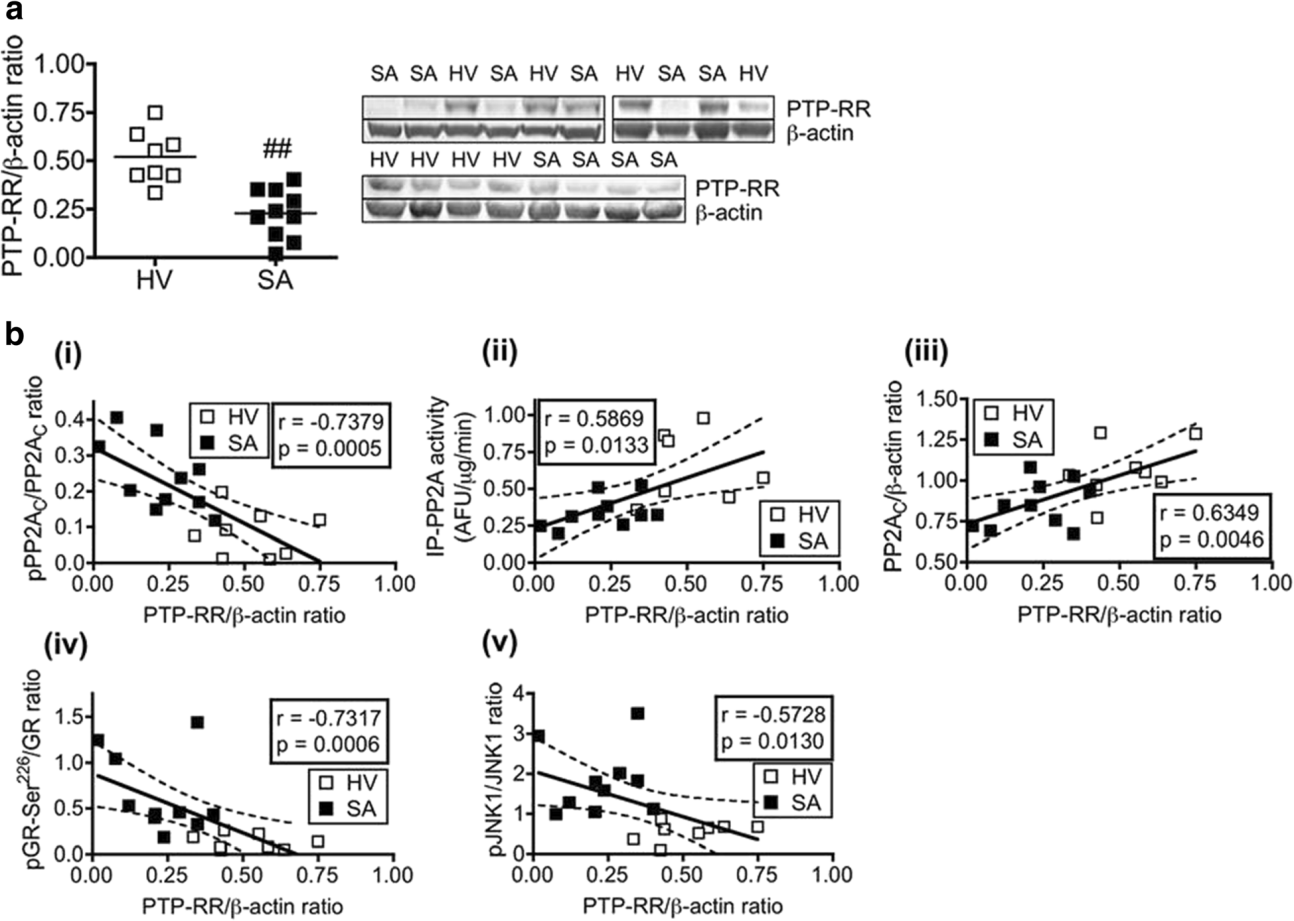
PTP-RR protein expression and its association with PP2A function and phosphorylation levels of GR and JNK1 in PBMCs. **a** PTP-RR expression in PBMCs from healthy volunteers (HV) and severe asthmatics (SA). Individual values and means of eight (HV) and ten (SA) subjects are shown. ^##^ *P* < .01 (vs. HV). **b** Correlation between PTP-RR protein expression and PP2A_C_-Tyr^307^ phosphorylation (i), IP-PP2A activity (ii), PP2A_C_ protein expression (iii), phosphorylation of GR-Ser^226^ (iv) and JNK1 (v) in PBMCs. Individual values (HV and SA) are shown and the dotted lines indicate 95% confidence interval

Consistent with the IL-2/IL-4-induced corticosteroid insensitive U937 model, PTP-RR expression in PBMCs from severe asthmatics was significantly lower than those from healthy volunteers (Fig. 4a). In line with the findings observed in PTP-RR KD study, PTP-RR expression levels in PBMCs from severe asthmatics and healthy volunteers negatively correlated with phosphorylation levels of PP2A_C_-Tyr^307^ and positively correlated with PP2A activity and expression (panel (i) to (iii) in Fig. 4b). Previously, we have shown that GR from severe asthmatic patients is hyperphosphorylated at Ser^226^ and associated with increased JNK1 phosphorylation [13]. Interestingly, we found in the present study that PTP-RR expression levels in PBMCs were negatively correlated with phosphorylation levels of GR-Ser^226^ and JNK1, suggesting an association between PTP-RR and the GR/JNK1 complex (panel (iv) and (v) in Fig. 4b). Regarding PTP-RR activity in PBMCs, we could not collect enough samples from patients because a lot of PBMCs were needed to detect the phosphatase activity in IP-PTP-RR. We confirmed that co-stimulation with IL-2/IL-4 reduced IP-PTP-RR activity in PBMCs from healthy volunteers (by up to 30 %) as observed in U937 cells (data not shown).

### PTP-RR is activated by formoterol

**Fig. 5:**
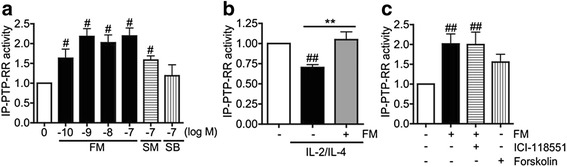
Effect of formoterol on immunopurified PTP-RR activity in U937 cells. **a** Cells were treated with formoterol (FM; 10^−10^-10^−7^ M), salmeterol (SM; 10^−7^ M) or salbutamol (SB; 10^−7^ M) for 20 min. **b** Cells stimulated with IL-2/IL-4 for 48 h were treated with FM (10^−9^ M) for 20 min. **c** Cells were preincubated with or without ICI-118551 (ICI; 10^−5^ M), a selective β_2_-adrenoceptor antagonist for 30 min, followed by treatment with FM (10^−9^ M) or cAMP inducer, forskolin (10^−5^ M). Data are expressed as fold change against non-treatment control (NT). Values represent means of four (**a** and **b**) or three (**c**) experiments ± SEM: ^#^ *P* < .05, ^##^ *P* < .01 (vs. NT), and ** *P* < .01 (as shown between two groups)

We have previously shown that a LABA, formoterol, restores corticosteroid sensitivity in severe asthmatics via restoration of PP2A activity [18]. Interestingly, formoterol also significantly enhanced PTP-RR activity (more than 2-fold at the concentrations of 10^−9^ to 10^−7^ M) (Fig. 5a). Furthermore, formoterol (10^−9^ M) completely reversed IL-2/IL-4-induced reduction of PTP-RR activity (Fig. 5b). These findings are supported by our previous study in which formoterol (10^−9^ M) restored corticosteroid sensitivity with submaximal effect (90 %) in the presence of IL-2/IL-4 [18]. Formoterol-mediated PTP-RR activation was not inhibited by a selective β_2_-adrenoceptor antagonist (ICI-118551), and PTP-RR activity was not enhanced by cAMP inducer, forskolin. The appropriate concentration of ICI-118551 (10^−5^ M) was determined by its inhibitory effect on formoterol-induced cAMP production (more than 90 %) [18]. In addition, PTP-RR was activated to a higher extent by formoterol (10^−9^ M) than by salmeterol (10^−7^ M), while salbutamol (10^−7^ M) had no effect (Fig. 5a and c). Importantly, this results denoted the same tendency as previous findings that formoterol (10^−9^ M) restored corticosteroid sensitivity in severe asthmatics with higher extent compared to salmeterol (10^−7^ M) [5]. Taken together, it seems that PTP-RR is activated by LABAs (especially formoterol) in a β_2_-adrenoceptor-independent fashion as recently reported with PP2A [18] and that PTP-RR regulates PP2A function.

## Discussion

**Fig. 6:**
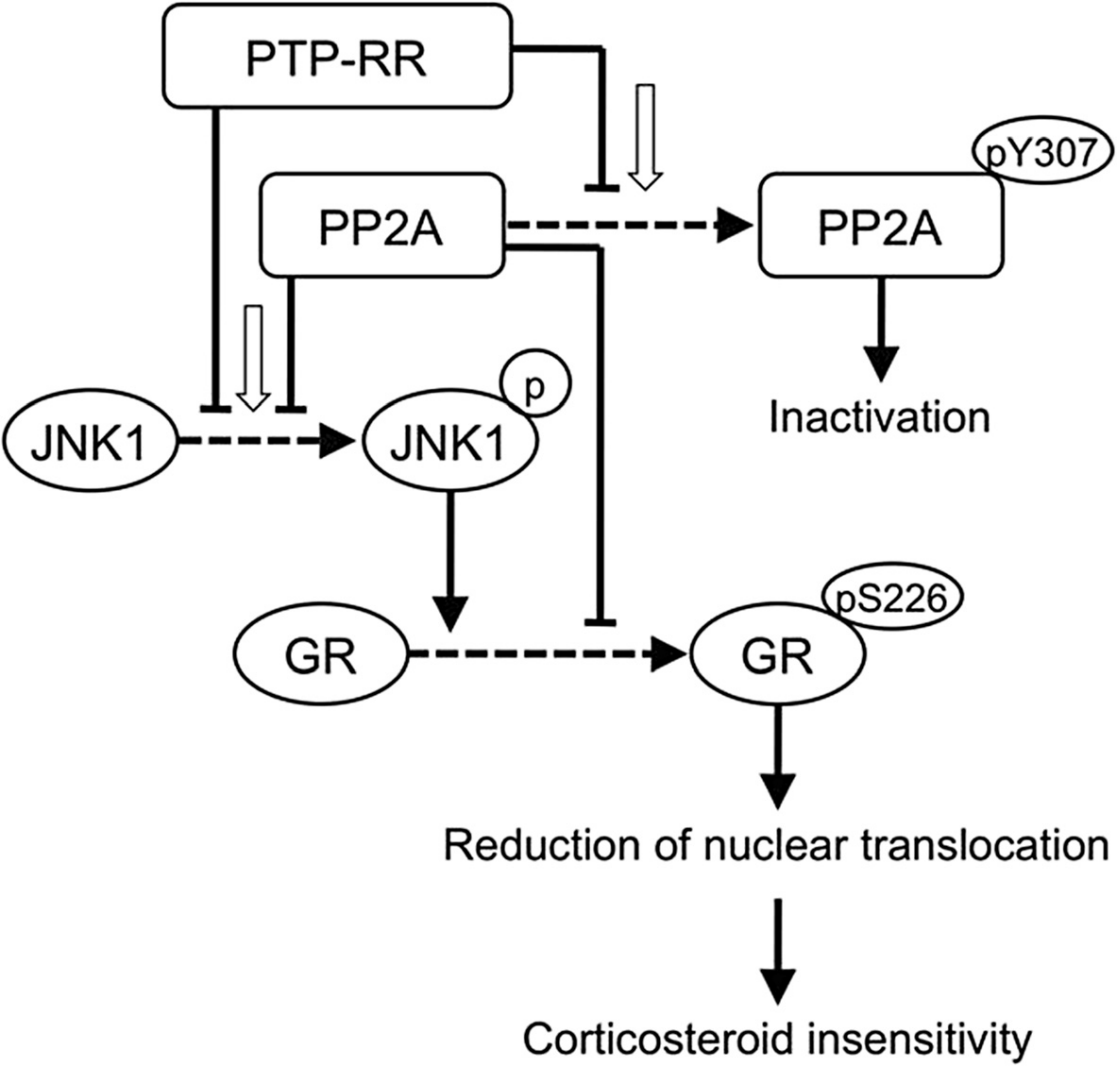
Proposal mechanism of regulation of corticosteroid sensitivity. Reduction of GR nuclear translocation by phosphorylation of GR-Ser^226^ is one of key mechanisms of corticosteroid insensitivity. Severe allergic inflammation associated with production of IL-2 and IL-4 (*white arrows*) induces phosphorylation of GR-Ser^226^ by JNK1 activation and PP2A inactivation (Tyr^307^ phosphorylation). PTP-RR regulates corticosteroid sensitivity by dephosphorylation of JNK1/GR-Ser^226^ directly and/or indirectly via PP2A

Our study has revealed a novel role for PTP-RR in the regulation of corticosteroid sensitivity by modulating GR nuclear translocation after corticosteroid treatment. Importantly, we confirmed that PTP-RR is down-regulated in PBMCs from severe asthmatics and in an *in vitro* corticosteroid insensitive cellular model induced by co-stimulation with IL-2/IL-4. In this model, PTP-RR was dissociated from GR/JNK1 complex leading to hyperphosphorylation of JNK1 and GR-Ser^226^. In addition, we found that impaired PTP-RR causes down-regulation of PP2A, which is also involved in JNK1 and GR-Ser^226^ phosphorylation by favoring of PP2A_C_-Tyr^307^ phosphorylation. Thus, it seems that PTP-RR might regulate corticosteroid sensitivity both directly and indirectly via modulation of PP2A activity (Fig. 6).

Reduction of GR nuclear translocation is one of causes of corticosteroid insensitivity in severe asthma [2, 4]. In fact, dexamethasone-induced translocation of GR into the nucleus is reduced in PBMCs from patients with severe asthma and this correlates with other indices of defective corticosteroid sensitivity in these patients [6]. Also in IL-2/IL-4-induced corticosteroid insensitive model, reduced GR nuclear translocation has been reported [4, 23]. We demonstrated that knocking-down PTP-RR reduces GR nuclear translocation with concomitant induction of corticosteroid insensitivity, and that PTP-RR expression was reduced in severe asthma and in the IL-2/IL-4-induced corticosteroid insensitive model. Our results suggest that PTP-RR might be a key molecule involved in the regulation of GR nuclear translocation in severe asthma.

Inhibition of PTP-RR by knockdown and co-immunoprecipitation studies indicated that PTP-RR is present in a GR/JNK1 complex and associated with dephosphorylation of GR-Ser^226^ and JNK1. We also examined the involvement of other MAPK-PTPs, PTP-N5 and PTP-N7 in phosphorylation of JNK1 and GR-Ser^226^. In U937 cells that were knocked down for PTP-N5 or PTP-N7, the phosphorylation levels of JNK1 were also enhanced, however neither PTP-N5 nor PTP-N7 knock-downs increased GR-Ser^226^ phosphorylation. SP600125, a JNK inhibitor partially reduced phosphorylation of GR-Ser^226^ in IL-2/IL-4-induced corticosteroid insensitive model [18], suggesting that other signals (kinases and/or phosphatases) may be involved in PTP-RR-mediated regulation of GR-Ser^226^ phosphorylation.

It has been reported that PTP-RR inactivates extracellular signal-regulated kinase (ERK)1/2 by dephosphorylation of tyrosine residues [25] and that PTP-RR associates with ERK1/2 and p38 through a kinase interaction motif (KIM) required for phosphorylation of PTP-RR by these kinases [26]. However, it was not previously established whether PTP-RR could dephosphorylate JNK1. We have recently found that a serine/threonine phosphatase, protein phosphatase 2A (PP2A) also dephosphorylates JNK1 and GR-Ser^226^ [13]. As we have shown an association between PTP-RR and PP2A (Fig. 3a), PTP-RR might dephosphorylate JNK1 and GR-Ser^226^ in collaboration with PP2A. Another possibility is that PTP-RR indirectly regulates phosphorylation levels of JNK1 and GR-Ser^226^ via PP2A. PP2A catalytic subunit, a regulator of PP2A complexes and activity [27], is phosphorylated at Tyr^307^ residue [28], resulting in reduction of PP2A activity [24]. We confirmed that increased phosphorylation of PP2A_C_-Tyr^307^ after knockdown of PTP-RR, but not of PTP-N5 and PTP-N7 leads to a decrease in PP2A activity (Fig. 3b, c, and Additional file 1: Figure S1). On the other hand, PP2A knockdown did not affect PTP-RR activity, expression (Fig. 3e and f) and association with JNK1 (data not shown), suggesting that PTP-RR could be associated with JNK1, independently of PP2A. Thus, PTP-RR, but not PTP-N5 and PTP-N7 regulates dephosphorylation of JNK1 and GR-Ser^226^ directly and/or indirectly via PP2A. Regarding the mechanism of PTP-RR down-regulation, MG-132, a proteasome inhibitor, abrogated IL-2/IL-4-induced reduction of PTP-RR protein expression, suggesting that a reduction in PTP-RR protein stability in severe asthma could be taking place. Recently, several studies demonstrated Tyr^307^ phosphorylation of PP2A is regulated via protein tyrosine phosphatase 1B (PTP1B) [29, 30]. Together with our findings, the conclusion can be drawn that several phosphatases with the ability to dephosphorylate tyrosine residues might control cellular functions in collaboration with PP2A.

PTP-RR is expressed predominantly in brain, and regulates cerebellar neurons and motor coordination [31]. In addition, PTP-RR has been also detected in other non-neuronal tissues, such as cartilage and intestinal mucosa, and regulates bone morphogenesis and tumor genesis, respectively [32, 33]. Importantly, it seems that PTP-RR negatively regulates tumor progression in neoplastic disorders such as cervical cancer or colorectal cancer [32, 34]. We have revealed for the first time that PTP-RR is also expressed in PBMCs and the U937 human monocytic cell line, and shows reduced expression in severe asthma and IL-2/IL-4-induced corticosteroid insensitivity, respectively. This suggests that PTP-RR might be a potential novel therapeutic target for various diseases with reduced corticosteroid sensitivity including severe asthma. PTP-RR was activated by formoterol although this effect was independent of the β_2_-adrenoceptor function. We have previously observed that formoterol was able to restore PP2A activity independently of β_2_-adrenoceptor, however the mechanism of action remains unknown [18]. Furthermore, as LABAs are reported to provide beneficial effects in exacerbation of allergic airway disease by activating PP2A [35], PTP-RR activation by LABAs may also contribute to their beneficial effects on asthma control.

## Conclusions

We have reported for the first time that PTP-RR is reduced in severe asthmatics and that in corticosteroid insensitive condition, impaired PTP-RR causes phosphorylation of JNK1 and GR-Ser^226^ directly and/or indirectly via PP2A, resulting in less corticosteroid-induced nuclear translocation of GR. Thus, PTP-RR is a novel therapeutic target for the restoration of corticosteroid sensitivity in severe asthma.

## Additional file

Additional file 1: Figure S1. Effect of PTP-N5 and PTP-N7 knock-down on regulation of PP2A. A, B, PP2A_C_-Tyr^307^ phosphorylation (A) and PP2A activity (B) were analyzed in U937 cells transfected with scramble control (SC), PTP-N5 (N5) and PTP-N7 (N7) siRNAs. Values represent means of three experiments ± SEM. (JPG 30 kb)

## Abbreviations

Ab: antibody
GR: glucocorticoid receptor
JNK: c-Jun N-terminal kinase
KD: knock-down
MAPK: mitogen-activated protein kinase
MKP: mitogen-activated protein kinase phosphatase
PBMC: peripheral blood mononuclear cells
PTP: protein tyrosine phosphatase
PP2A: protein phosphate 2A
