# Supplementary figures and images for: Protein tyrosine phosphatase PTP-RR regulates corticosteroid sensitivity
Source: Respir Res. 2016 Mar 24;17:30. doi: 10.1186/s12931-016-0349-0 (PMC4806463; doi:10.1186/s12931-016-0349-0)

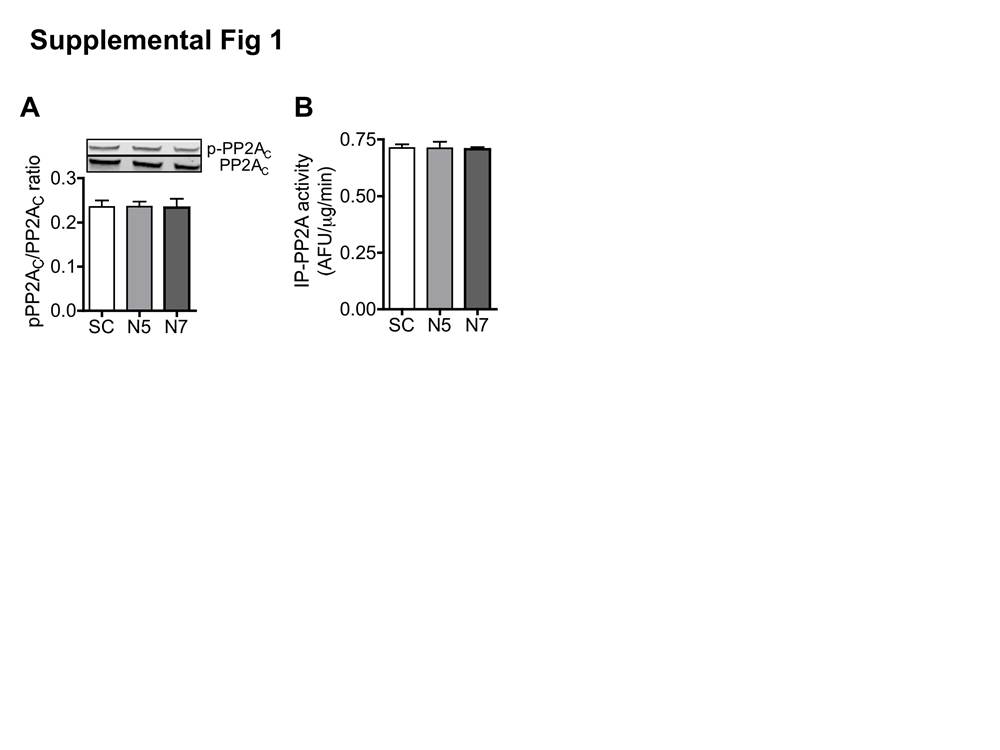

Supplement: Additional file 1: Figure S1. — Effect of PTP-N5 and PTP-N7 knock-down on regulation of PP2A. A, B, PP2AC-Tyr307 phosphorylation (A) and PP2A activity (B) were analyzed in U937 cells transfected with scramble control (SC), PTP-N5 (N5) and PTP-N7 (N7) siRNAs. Values represent means of three experiments ± SEM. (JPG 30 kb) [file 12931_2016_349_MOESM1_ESM.jpg]
